# Interval exercise versus continuous exercise in patients with moderate to severe chronic obstructive pulmonary disease – study protocol for a randomised controlled trial [ISRCTN11611768]

**DOI:** 10.1186/1471-2466-4-5

**Published:** 2004-08-13

**Authors:** Milo A Puhan, Gilbert Büsching, Evelien vanOort, Christian Zaugg, Holger J Schünemann, Martin Frey

**Affiliations:** 1 Horten Centre, University of Zurich, Switzerland; 2 Klinik Barmelweid, Barmelweid, Switzerland; 3 Experimental Cardiology Research Group, Dept. Research, University of Basel, Switzerland; 4 Department of Clinical Epidemiology and Biostatistics, McMaster University, Hamilton, Ontario, Canada; 5 Departments of Medicine and of Social & Preventive Medicine, University at Buffalo, Buffalo, New York, USA

## Abstract

**Background:**

Physical exercise has become a cornerstone of management of chronic obstructive pulmonary disease (COPD) because it leads to clinically relevant improvements of exercise capacity and health-related quality of life (HRQL). Despite the scarcity of randomised trials directly comparing exercise protocols, current guidelines recommend high intensity continuous exercise for lower extremities as the probably most effective exercise modality. However, for patients admitted to inpatient respiratory rehabilitation programmes, it is often difficult to initiate such an exercise programme because they are severely limited by dyspnoea and leg fatigue and therefore unable to perform continuous exercise at higher intensities and for periods longer than 30 minutes. Interval exercise may be an attractive alternative for these COPD patients because it allows high intensity exercise with recovery periods. The aim of this study is to assess if interval exercise compared to high intensity continuous exercise is not of inferior effectiveness in terms of HRQL and exercise capacity improvements but associated with better exercise tolerance in patients with moderate to severe COPD at the beginning of a respiratory rehabilitation.

**Methods/Design:**

We will assign patients with moderately severe to severe COPD to either continuous exercise or interval exercise using a stratified randomisation. Patients will follow 12–15 exercise sessions during a comprehensive inpatient respiratory rehabilitation. Primary end point for effectiveness is HRQL as measured by the Chronic Respiratory Questionnaire (CRQ) two weeks after the end of rehabilitation and secondary endpoints include additional clinical outcomes such as functional exercise capacity, other HRQL measures, patients' experience of physical exercise as well as physiological measures of the effects of physical exercise such as cardiopulmonary exercise testing. Including expected drop-outs, we will need 52 patients per group to show differences corresponding to the minimal clinically important difference of the CRQ. Outcome assessors and investigators involved in data analysis will be blinded to group assignment until analyses have been carried out.

**Discussion:**

Clinicians and the scientific community need evidence on the benefits and tolerance of exercise protocols available in clinical practice. The proposed trial will provide important and needed data on interval and continuous exercise for decision making in clinical practice.

## Background

Impaired exercise capacity, dyspnoea and reduced health-related quality of life (HRQL) are common complaints of patients with chronic obstructive pulmonary disease (COPD). A major exercise-limiting factor in COPD is peripheral muscle dysfunction characterised by atrophic muscles and reduced fatigue resistance due morphological and metabolic alterations of peripheral muscles[1] As much as 70% of COPD patients may be affected by peripheral muscle dysfunction.[2]

Respiratory rehabilitation with physical exercise improves exercise capacity and HRQL.[3] Although physical exercise is a mandatory component of respiratory rehabilitation programmes[4,5], there is an ongoing debate about what type of exercise at which exercise intensity patients should perform.[1,6] There is substantial variation in exercise protocols used in practice[7] as well as in clinical trials[3]. Current guidelines recommend continuous exercise at high intensity for lower extremities[4,5] because a study indicated that high intensity may be more effective than low or moderate intensity.[8]

However, data on high intensity continuous exercise come from a trial that included 19 patients with mild COPD who were able to exercise for 45 minutes five times per week during an outpatient programme.[8] For patients who need to be admitted to inpatient programmes because of more severe COPD and/or unstable health state, it is difficult to perform high intensity exercise and exercise sessions longer than 30 minutes because they are limited by dyspnoea and leg fatigue. Less than 20% may be able to sustain high intensity continuous exercise throughout the whole rehabilitation programme[9] To find a realistically tolerable exercise programme for these patients, who often initiate exercise programmes for the first time, is challenging.

A solution to this dilemma may represent interval exercise[6] where patients exercise alternatively at high intensity and at low intensity, which allows short periods of recovery. Consequently, interval training may be better tolerated than high intensity continuous training. In addition, patients may be able to achieve a greater training load during the relatively short exercise sessions they can sustain.

Patients and clinicians will accept interval exercise to treat peripheral muscular dysfunction in COPD only if it is not of inferior effectiveness compared with continuous exercise and if it is indeed associated with better compliance resulting from less dyspnoea and leg fatigue during exercise. There is limited evidence from three randomised controlled trials comparing interval exercise and continuous exercise.[10-12] A summary of these trials can be found in table 1.

**Table 1:** Trials on interval exercise in patients with COPD

	**Population**	**Exercise protocols and rehabilitation program**	**Main results**
Coppoolse 1999 [10]	21 stable male COPD patients (mean age 65 years, FEV1 36.8% predicted)	**Group 1**: CT ergometer cycling at 60% of Wmax **Group 2**: IT ergometer cycling at 90% of Wmax (1 min) and 45% of Wmax (2 min) 3 days/week plus CT ergometer cycling at 60% of Wmax 2 days/week 8 weeks inpatient rehabilitation with 5 exercise sessions per week of 30 min. No additional physical exercise.	Significant increase of V_O2 _and decrease of minute ventilation with CT but no changes with IT. Significant increase of Wmax and decrease of leg pain during exercise with IT but not with CT. Only significant differences between CT and IT for V_O2_/Wmax favouring CT. 91% of patients with CT and 90% of patients with IT completed the exercise program.
Vogiatzis 2002 [11]	45 stable COPD patients (62% males, (mean age 65 years, FEV1 34.1% predicted)	**Group 1**: CT ergometer cycling at 50% of Wmax weeks 1–4, at 60% weeks 5–8 and at 70% weeks 9–12 **Group 2**: IT ergometer cycling at 100% of Wmax (30 sec) and 45% of Wmax (30 sec) weeks 1–4, at 120% weeks 5–8 and at 140% weeks 9–12 12 weeks outpatient rehabilitation with 2 exercise sessions per week of 40 min. No additional physical exercise.	Significant improvements of CRQ scores and Wmax and reductions of minute ventilation during CWRT in both groups. No significant differences between groups. Attendance rate for exercise sessions 88% for CT and 90% for IT.
Kaelin 2001 [12]	19 stable COPD patients (89% males) (mean age 67 years, FEV1 26.9% predicted)	**Group 1**: CT walking on stepper (70 steps/minute) or treadmill (1.5 miles/hour). Increase of 1 MET every 2 weeks **Group 2**: IT walking on stepper (70 steps/minute) or treadmill (1.5 miles/hour) with active rest to ratio of 2:1. Increase of 1 MET every 2 weeks 6 weeks outpatient rehabilitation with 3 exercise sessions per week of 10–30 min. Additional resistance training and flexibility training.	Larger improvements of 6-minute walking distance with IT (80 meters) compared with CT (39 meters). No data on compliance.

CT = Continuous training; IT = Interval training; CRQ = Chronic Respiratory Questionnaire Wmax = Maximum exercise capacity, measured by usual incremental exercise test; CWRT = Constant work rate test; V_O2 _= Maximum oxygen consumption MET = Metabolic equivalent

The studies indicated that both interval and continuous training improved exercise capacity, dyspnoea and HRQL and showed insignificant differences between interval and continuous exercise. However, insignificant differences do not allow concluding that interval or continuous are of clinically equivalent effectiveness[13] These trials were too small to show clinical equivalence or non-inferiority and they did not provide evidence on the tolerance of these two exercise modalities. From a methodological point of view, the trial had several shortcomings because, for example, they did not provide details on concealment of random allocation or blinding of outcome assessors. In addition, in the trial with an inpatient rehabilitation.[10], patients of the interval exercise group had a mixed intervention (3 days of interval and 2 days of continuous exercise per week) so that differences can hardly be attributed to different interventions if they are detected at all.

The investigators did not use steep ramp tests to determine exercise loads but normal incremental exercise tests. For interval exercise, muscle strength and anaerobic capacity is relevant because of the short high intensity intervals, but this is not measured by normal incremental exercise tests. In addition, training load tolerated during interval exercise may be underestimated when normal incremental exercise tests are used.[14]

Exercise tests to establish training intensity should consider the exercise mode. Meyer et al. studied interval exercise in several studies [14-16] in patients with chronic heart failure who show similar patterns of physical deconditioning in terms of clinical manifestation as well as morphological and metabolic abnormalities [17-19] Meyer et al. used a steep ramp test to determine short time muscular maximum exercise capacity[14], which reflects muscle strength and anaerobic capacity, both relevant for interval exercise.

Meyer et al. also assessed different ratios of work/recovery phases (1:2; 1:4 and 1:6) and found that with these ratios and relatively short phases of high intensity exercise (10–30 seconds), lactate did not accumulate presumably because of lactate elimination during the recovery phases.[14] Interval exercise with these ratios was therefore recommended as high intensity aerobic exercise modes for patients who do not sustain continuous exercise.

Because of the scarcity of evidence on the comparative effectiveness of interval exercise for COPD patients, additional trials are needed.[1,6] Our primary objective is to assess if interval exercise is not of inferior effectiveness compared to continuous exercise of high intensity to improve HRQL and exercise capacity in patients with moderately severe to severe COPD and the secondary objective is to evaluate if interval exercise is better tolerated by COPD patients.

## Methods

### Study design (see figure 1)

**Figure 1:**
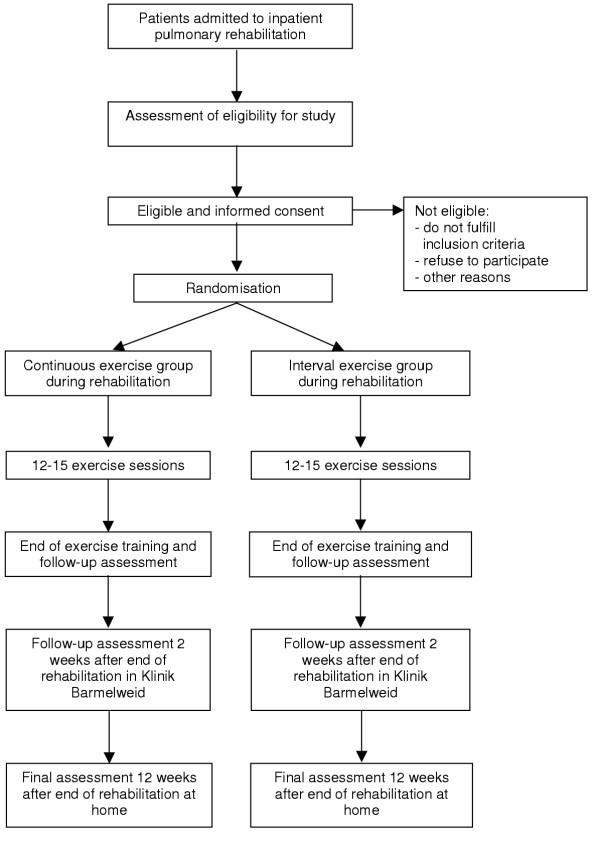
Flow of the study from screening for eligible patients to the final outcome assessment.

All consecutive patients admitted to a teaching rehabilitation clinic for an inpatient respiratory rehabilitation (Klinik Barmelweid, Barmelweid, Switzerland) will be assessed for study eligibility by senior staff physicians. If patients are deemed eligible after exercise testing, which is part of the usual rehabilitation program, senior physicians will inform patients about the study orally and in writing. If patients are willing to participate and provide written informed consent they will be randomly assigned to respiratory rehabilitation with either interval or high intensity continuous exercise. Both groups will perform 12–15 exercise sessions and follow the rest of the rehabilitation programme. Follow-up assessments will be done during, at the end of the rehabilitation programme as well as two and twelve weeks afterwards when patients are back in their home environment.

The Ethikkommission of the Kantonsspital Aarau, Aargau, Switzerland, has approved the study protocol.

### Patients

We defined the following in- and exclusion criteria: Patients with COPD as defined by FEV1/FVC < 70% predicted, FEV1 < 50 % predicted after bronchodilation, with or without chronic symptoms (cough, sputum production) corresponding to a GOLD (Global Initiative for Chronic Obstructive Lung Disease) stage III-IV[20] and German as first or daily language. Exclusion criteria are arrhythmia (atrial flutter and fibrillation, ventricular tachycardia, premature beats > 8 per minute), ischemia during exercise testing, clinically decompensated Cor pulmonale or heart failure, untreated neoplasia or neoplasia that needed treatment within the previous two years, lung surgery within the previous three months, orthopedic, rheumatologic, vascular or neurological disorders that inhibit ergometer training, gymnastic or guided walking tours, and patients unable to perform or complete the six-minute walk test or the incremental cycle test

### Randomisation

A third party not involved in the conduction of the trial will provide online central randomisation (DatInf GmbH, Tuebingen, Germany). A computerised 'minimisation' procedure will be used to avoid chance baseline imbalances in prognostically important variables[21] Minimisation variables will be exercise capacity (< 300 or ≥ 300 meters in six-minute walk test), the presence of affective disorders (Hospital Anxiety Depression Scale scores < or ≥ 8), status of COPD (unstable COPD = In- or outpatient medical care in the last eight weeks due to exacerbation of COPD versus stable COPD = no in- or outpatient medical care in the last eight weeks due to exacerbation of COPD) and the need for oxygen at rest (yes = long term home oxygen therapy or paO_2 _< 55 mmHg or no = paO_2 _≥ 55 mmHg).

Every time the study coordinator has been informed about an enrolled patients, he will enter the randomisation web site[22] enter the patient data required for randomisation (patient identification and stratification variables) and obtain group allocation. The study coordinator will then inform responsible physical therapists about group allocation. No other medical staff will have knowledge about group allocation. The randomisation provider will also send an e-mail to the study coordinator for each randomised patient with details on randomisation. This will ensure correct verification of group allocation after data analysis.

Separating patient enrolment and baseline assessments (physicians and physical therapists) from the randomisation procedure (study coordinator not involved in patient enrolment or rehabilitation programme) will ensure concealment of random allocation.

### Interventions

The rehabilitation programme will start one day after baseline assessments and study enrolment. Exercise sessions and group lessons will take place five days a week and will consist of daily cycle ergometer training, breathing therapies (30 minutes per day), and guided walking of 15 to 30 minutes. Relaxation therapies (technique according to Jacobson) take place twice a week, patient education (information about COPD, coping strategies, inhalation techniques) three times a week, smoking cessation advice once a week or more if needed. Apart from physical exercise, the rehabilitation programme of approximately three weeks will be identical for both groups.

#### Group performing continuous exercise

**Figure 2:**
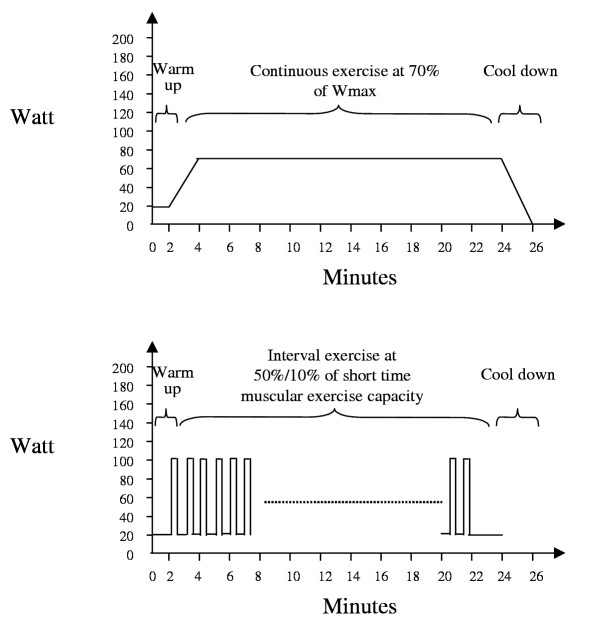
The upper graph shows the continuous exercise protocol for a patient who achieved a maximum exercise capacity of 100 Watts during a usual incremental exercise test. The lower graph shows the interval exercise protocol for a patient who achieved a short time muscular exercise capacity of 200 Watts during a steep ramp test.

The target workload for this group will be ≥ 70% of the maximum exercise capacity expressed in Watts and heart rate achieved during the incremental cycle ergometer test. Patients are usually not able to perform high intensity continuous exercise from the beginning and have to adapt to physical exercise. Physical therapists will increase training load as soon as possible to ≥ 70% of the maximum exercise capacity or as high as each individual patient tolerates. In each session (see figure 2), patients will have a warm-up period of two minutes at 20% of maximum exercise capacity, increase the exercise intensity within two minutes to the target intensity, exercise for 20 minutes at high intensity and then have a decreasing period of two minutes (gradual decrease from 70% to 0%). Pulse oxymetry will be used to supervise patients during exercise. If oxygen saturation falls below 90%, oxygen supplementation will be provided to maintain ≥ 90%.

If patients cannot sustain the workload because of perceived dyspnoea or leg fatigue or because the heart rate exceeds the limits determined during exercise testing, physical therapists will let patients rest for one minute and then resume exercise. If patients have to rest more than twice per session, physical therapists will lower the workload by steps of 10% of baseline maximum exercise capacity. In turn, if patients or physical therapists consider the workload to be too low or if patients do not reach their target heart rate at 70% of the maximum exercise capacity, physical therapists will increase workload by steps of 10% of maximum exercise capacity until patients and physical therapists consider the workload to be appropriate or until the target heart rate is reached.

#### Group performing interval exercise

Patients assigned to this group will perform a steep ramp test to determine the short time muscular maximum exercise capacity.[14] The steep ramp test is an incremental cycle ergometer test where patients pedal unloaded for 2 minutes and then pedal with increments of 25 Watts every 10 seconds until they cannot maintain a pedaling frequency above 50 per minute or above the heart rate limit set by the normal incremental exercise test (which all patients perform irrespective of group assignment). With the steep ramp test measurement of muscular maximum exercise capacity is possible because the tests lasts only for 30 to 120 seconds and patients are not limited by symptoms. Measuring muscular maximum exercise capacity is important to set the exercise load for interval exercise because the high intensity interval requires also muscle strength beside overall exercise endurance.[14]

Patients should improve both endurance and muscle strength during interval exercise because both are required in daily activities. We therefore chose a work/recovery ratio of 1:2 that prevents from high lactate accumulation.[14] From short time maximum exercise capacity, we will derive the initial work rate for interval exercise (in Watts), which is set at 50% of the short time muscular maximum exercise capacity as measured by the steep ramp test. This workload corresponds in Watts approximately to 90–100% of the workload as measured by the normal incremental exercise test.[16].

Patients will start with exercise the day after the steep ramp test. Patients will perform interval exercise for twelve to fifteen sessions with a cycle ergometer. In each session, they will have a warm up period of two minutes at 20% of the short time maximum exercise capacity (figure 2). Then they exercise for 20 minutes at high intensity intervals of 20 seconds at 50% and at low intensity intervals of 40 seconds at 20% of the short time maximum exercise capacity, i.e. with a work/recovery ratio of 1:2. Then patients have a slow down period of two minutes before completion of the training session. Pulse oxymetry will be used and oxygen supplementation will be provided as described above.

If patients cannot sustain exercise intensity because the heart rate exceeds the limits determined after exercise testing or because of perceived dyspnoea or leg fatigue, physical therapists will let patients rest for one minute and then resume exercise. If patients have to rest more than twice per session, physical therapists will lower the workload from 50% of the short time maximum exercise capacity by steps of 10% while the length of intervals remains constant. They will increase the training load again as possible for the patient.

In turn, if patients or physical therapists consider the workload to be too low, physical therapists will increase workload of the high intensity interval by steps of 10% until patients and physical therapists consider the workload to be appropriate while the length of intervals remains constant.

### Clinical outcome measures

#### Chronic Respiratory Questionnaire (CRQ)

We will use the CRQ[23] to measure HRQL changes during rehabilitation. The CRQ is a widely used disease-specific instrument to assess symptoms of COPD patients [24-26] We will use the self-administered German CRQ.[27,28] with standardised dyspnoea questions.[29] that we have developed and validated in earlier studies. Patients will complete the CRQ in the Klinik Barmelweid at baseline, at the end of the rehabilitation, two and 12 weeks thereafter when they have returned to their home environment.

#### HADS (Hospital Anxiety Depression Score)

Affective disorders are common in patients with COPD and contribute to reduced HRQL[30] The HADS has been developed to assess symptoms of anxiety and depression in patients with physical impairment.[31] There are seven items for each domain (anxiety and depression) with statements on emotions and emotional situations. Patients express their agreement with the statements on a scale from 0 to 3. Domain scores are calculated by summing up the scores for the seven domains resulting in scores from 0 (no depression or anxiety) to 21 (depression or anxiety very likely to be present). Scores ≥ 8 indicate that there is an increased probability for the presence of an affective disorder. We will use the validated self-administered German version of the HADS.[32] The HADS will be completed in the Klinik Barmelweid at baseline, at the end of the rehabilitation, two and 12 weeks thereafter when they have returned to their home environment.

#### Feeling Thermometer (FT)

We will use a validated visual analogue scale, the FT[33], an increasingly used instrument for a global estimate of the effect of interventions, including respiratory rehabilitation[29,34]. The FT is a visual analogue scale presented as a thermometer with 100 marked intervals. The worst (dead = 0) and best (perfect health = 100) health states are defined anchors and facilitate comparisons between individuals and groups.[35] We will ask patients to reflect in their score how they felt in the last 7 days.

The FT will be completed in the Klinik Barmelweid at baseline, at the end of the rehabilitation, two and 12 weeks thereafter when they have returned to their home environment.

#### Six-minute walk test

We will use the six-minute walk test to assess functional exercise capacity according to established criteria.[36] At baseline, patients will perform the six-minute walk test twice one day apart. We will use the results of the second six-minute walk test because the first test tends to underestimate exercise capacity due to unfamiliarity with the test.[36]. At the end of the rehabilitation and two weeks after completion of rehabilitation programme patients will perform additional six-minute walk tests under supervision of physical therapists blinded to group assignment. In addition, we will use a paperboard with a modified Borg scale on from 0 to 10 with verbal labels for 0 (no dyspnoea at all), 1–5, 7 and 10 (maximal dyspnoea) to assess the intensity of perceived dyspnoea at the end of the six-minute walk test.

### Monitoring of exercise sessions

#### Dyspnoea and leg pain during exercise

In each session, we will use modified Borg scale as described above to assess the intensity of perceived dyspnoea and leg pain after five minutes of exercise and at the end of the exercise sessions.

#### Subjective experience of exercise

There is no instrument available to assess the subjective experience of COPD patients with exercise, which is likely to influence compliance. We have developed a questionnaire using established methodology[37] to assess how patients experienced the sessions. The questionnaire (Exercise Tolerance Questionnaire) consists of five questions addressing the exercise limitation by shortness of breath and difficulties with breathing, leg fatigue, fatigue in general and too high exercise load. In addition, one question asks patients how they experienced the exercise session in general (from very enjoyable to very unpleasant). The report on questionnaire development will be published elsewhere.

#### Adherence to and tolerance of the prescribed cycle ergometer exercise

Physical therapists will record for every cycle ergometer exercise session if patients exercised at all (yes or no), the performed workload (in Watts), if patients reached the target workload (in Watts), adjustments of workload and the requirement for oxygen. We define adherence to exercise as "full adherence" if patients follow at least 12 exercise sessions. We consider the training to be fully tolerated if patients are able to follow the exercise protocol for at least 9 exercise sessions for continuous exercise (taking into consideration the first three exercise session when patients increase training load up to 70% of maximum exercise capacity) and for at least 12 exercise sessions for interval exercise.

#### Adverse events

Previous trials of respiratory rehabilitation or physical exercise did not report any adverse events. Nevertheless, we will record any adverse events such as injuries, cardiac events or increase of respiratory symptoms as they happen during the inpatient respiratory rehabilitation.

### Cardiopulmonary exercise testing

At baseline and end of the rehabilitation, all patients will perform an incremental cycle ergometer test to the limit of tolerance (symptom based) under the supervision of a senior physician blinded to group assignment. Patients pedal unloaded for three minutes to warm up at 20 Watts. Then exercise load is increased by 7.5 Watts per minute until patients have to stop because of dyspnoea, leg pain, or criteria for stop testing (atrial or ventricular tachycardia, ischemia, hypoxemia). At the limit of tolerance, we will draw capillary blood samples to measures lactate concentrations and we will set the maximum exercise capacity expressed by Watts. The upper limit for the heart rate during exercise is set as the heart rate measured by the electrocardiogram at the maximum exercise capacity.

During testing, we will record gas exchange and ventilatory variables form calibrated signals derived from rapidly responding gas analyzers and a mass flow sensor. We will record breath by breath the following variables: Pulmonary oxygen uptake, pulmonary CO_2 _output, minute ventilation, tidal volume and respiratory frequency.

All patients will perform a steep ramp test at the beginning and end of the rehabilitation programme as described above.

#### Additional data to be collected

In order to characterize the patient included in the study, we will record their age, gender, lung function (FEV1, FEV1/FVC, diffusion capacity DLCO/VA, weight, height, smoking status at the beginning and end of rehabilitation as well as two and 12 weeks afterwards, duration of disease (time since diagnosis), co-morbidities such as hypertension, heart diseases, endocrine disorders, chronic rheumatological disorders and psychiatric disorders.

### Data analysis

The randomisation code will not be broken (investigators remain blinded to group assignment and will receive only codes, such as group 1 and 2) until a draft of the manuscript has been written. The authors will write two versions with alternative possible allocation patterns to avoid bias in the interpretation of the results. This approach is methodologically rigorous and limits introduction of bias during the interpretation of the data that some of the authors might have.[38] We will submit the appropriate manuscript regardless of the results of unmasking. After agreement on the final versions of the two articles, we will break the randomisation code and we will submit the correct version of the manuscript.

### Effectiveness

Our null hypothesis is that high intensity continuous exercise is of clinically superior effectiveness compared with interval exercise to improve HRQL (δ ≥ 0.5 in CRQ domains scores). The alternative hypothesis is that interval exercise is not of clinically inferior effectiveness compared with high intensity continuous exercise.

In the primary analysis, we will calculate the raw difference and 95% confidence intervals between groups in the mean follow-up score for the CRQ domains 2 weeks after completion of the respiratory rehabilitation programme. In an additional analysis, we will adjust for the base-line score and the four stratification variables using an analysis of covariance. We will use independent t-test to compare the change scores between groups.

**Figure 3:**
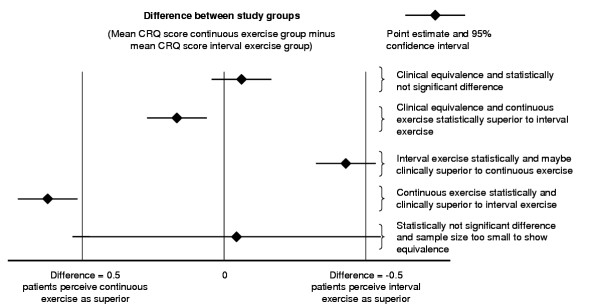
Illustration of the confidence interval approach to interpret results from randomised trials. The horizontal line indicates the difference between CRQ change scores between study groups. ± 0.5 points represent the predefined boundaries of equivalence. If the whole confidence interval is on the right of 0.5 points, interval exercise is not inferior to continuous exercise. If the whole confidence interval is within boundaries the two exercise protocols are of clinically equivalent effectiveness (upper three confidence intervals). Note that there can be a statistically significant difference between study groups but without any clinical relevance.

We will also use the confidence interval approach as recommended for equivalence and non-inferiority trials[13] We will establish non-inferiority of interval exercise if the point estimate and its 95% confidence interval for difference between the change scores of the continuous and interval exercise group is smaller than the a priori determined boundary of clinical equivalence (see figure 3). If the 95% confidence intervals lie outside the boundaries of clinical equivalence we will establish clinical superiority of one exercise protocol.

Most methodologists and statisticians agree that the boundaries of equivalence should be defined as the minimal important difference and below the differences observed in previous trials comparing active to control treatments.[13,39] The minimal important difference is "the smallest difference in score in the outcome of interest that informed patients perceive as important, either beneficial or harmful, and which would lead the clinician to consider a change in the management"[40]. Therefore, the minimal important difference of our two main outcome measures for effectiveness have been established empirically and are around 0.5 for the CRQ domains scores [41-44] and 53 meters for the six-minute walk test[45]. A recent meta-analysis showed that respiratory rehabilitation with physical exercise leads to improvements of 50 meters in the six-minute walk test. Therefore we lower the boundaries for equivalence to ± 45 meters in order to have boundaries that are below the differences observed in previous trials comparing active to control treatments.[13,39]

We will repeat the analysis with calculations of raw and adjusted differences for all other clinical and physiologic outcome measures and test for significant differences between groups using independent t-test if data are distributed normally.

We will use both intention to treat and per protocol analysis to show equivalence in either case as recommended by others[13,39,46,47]

### Exercise tolerance

Our null hypothesis is that patients equally experience high intensity continuous exercise and interval exercise as measured by the Exercise Tolerance Questionnaire. The alternative hypothesis is that interval exercise is associated with the experience of less limiting symptoms compared with high intensity continuous exercise.

We will use independent t-tests to compare the measures for exercise tolerance (Exercise Tolerance Questionnaire, Borg scales for dyspnoea and leg pain) between groups. Again, we will assess the raw differences between groups in the primary analysis and adjust for baseline scores and the four stratification variables in the additional analysis.

### Confounder variables and effect modifiers

Factors, which interfere with outcome measures, can distort the results if unevenly distributed between study groups. We use three approaches to control for confounders: First, we use randomisation to allocate patients. Second, we strengthen the randomisation using a computerised minimisation procedure with factors that are likely to influence the outcome measures (exercise capacity, pulmonary state and presence and absence of affective disorders). Third, we will collect a number of variables at baseline (age, gender, lung function, time since diagnosis, cumulative dose of oral steroids in previous three months, medication, cardiovascular, musculoskelettal and endocrine co-morbidities) that may modify the effect of exercise. We will compare the distribution of these potential effect modifiers between groups and statistically assess their influence on the outcome measures with multiple linear regression models.

#### Sample size

For calculating the required sample size, we use the formula for comparison of 2 means: n = [A + B]^2 ^* 2 * SD^2^/DIFF^2^, where n = the sample size required in each group (double for total sample), SD = standard deviation of the outcome variable, DIFF = size of desired difference between groups. A and B depend on the desired significance level and desired power, respectively.

We base our sample size calculations on the CRQ. We used empirical data from our previous trial.[27], where we applied similar inclusion criteria for patients with COPD undergoing respiratory rehabilitation, to estimate variability of the CRQ (standard deviation of the CRQ domain scores between 0.8 and 1.2). A clinically sensible way to determine the size of desired difference between groups (DIFF in formula above) is based on the minimal important difference (0.5 on the scale from 1 to 7 for the CRQ)[41-43,48] A sample size of 44 patients in each group will allow showing a difference of 0.5 in CRQ scores between the groups, assuming a standard deviation of 0.8, with a power of 90% at a significance level of 5% (one-sided). Assuming a drop out rate of 15%.[27], the total minimal sample size increases to 104. This sample size will also allow detecting a difference of 45 meters in the six-minute walking test scores between the two treatment groups with a power of 95% at a significance level of 5% (one-sided) assuming a drop out rate of 15%.

A priori sample size calculations usually provide only rough estimates. Therefore, we will recalculate sample size during the study when we have the data for 30 patients in each group. To this end, we will re-assess the standard deviation of the outcome variables and, if necessary, adjust the sample size accordingly (without breaking the randomisation code).

### Data collection and quality control

We will implemented a series of measures to ensure high data quality.

1. Site investigators will collect the data using standardized forms. All data will be collected and entered into a single database in the Horten Centre by one investigator. A second investigator will validate completeness and accuracy of data extraction by checking 20% of extracted patient data.

2. Checklists with all the data to be collected will be provided for physical therapists and physicians.

3. Teaching sessions will be held regularly for physical therapists and physicians involved in data collection aiming at robust data collection.

4. Investigators meetings will be held monthly, or more frequently if needed, to discuss recruitment of patients, problems in conducting the study, acquisition of data, to check for consistency and completeness of data and for interim analyses.

5. E-mail will serve as the first line of non-urgent communication between research team members.

6. Monthly reports will be prepared by the principal investigator that will include the number of patients recruited, stage of follow up for each patient, notification regarding missing patient data and queries from data received.

## Discussion

In the last 30 years, researchers made great efforts to study the effectiveness of respiratory rehabilitation compared to usual care. The meta-analyses of a recent systematic review[3] showed that respiratory rehabilitation with physical exercise leads to clinically significant improvements of HRQL as well as to significant improvements of functional and maximum exercise capacity.

Research in respiratory rehabilitation should now focus on the evaluation of different exercise protocols. When an effective treatment such as respiratory rehabilitation is available, patients and clinicians are not confronted with the decision whether to start treatment or not, but with the decision on the most appropriate treatment. Therefore, clinicians need evidence from pragmatic randomised controlled trials directly comparing different exercise protocols at issue rather than evidence from (explanatory) trials comparing exercise with no exercise or usual care.[49] The proposed pragmatic trial will therefore provide important and needed guidance for decision-making in respiratory rehabilitation, in particular for those COPD patients who are severely impaired and initiate respiratory rehabilitation programmes.

The evidence generated in the proposed clinical trial will also be relevant for the scientific community. From the 1970s up to the mid-1990s most investigators conducted explanatory clinical trials in order to better understand how and why physical exercise is effective in patients with COPD. After the recognition of its effectiveness the debate on the optimal exercise modality arose[6]. However, only few pragmatic trials have been conducted so far to advance the understanding of the relative benefit and downsides of different exercise protocols. With the proposed trial, we compare two clinically relevant interventions with the use of a clinical trial design that is useful for clinical decision making. Such trials are currently needed to gain consensus on the optimal exercise protocol.

There is a need for randomised controlled trials to explore which exercise protocols are most effective for COPD patients. Another topic that has received little attention in respiratory rehabilitation trials so far is compliance to and subjective experience of physical exercise[9] Exercise protocols and adherence to it have not been described in detail in published studies[3] and therefore very little is known about the tolerance of different training modalities. This is despite the fact that physical exercise has long been considered unfeasible in patients with COPD.

Beside the paucity of data on the effectiveness and tolerance of different exercise protocols, there is also a need for trials that are methodologically sound and rigorous. Most of the studies on exercise or respiratory rehabilitation in patients with COPD did not report on details of exercise tests and protocols. In addition, study design related issues that introduce bias such as description of the randomisation procedure, concealment of allocation, sample size calculations or blinding have not been addressed frequently. Therefore, we try to use strong epidemiological methods to minimize bias in our trial.

Our study could make an important contribution to the understanding of physical exercise in patients with COPD and have a significant impact on the structure of respiratory rehabilitation and exercise programmes.

## Competing interests

None declared.

## Abbreviations

COPD = Chronic obstructive pulmonary disease

HRQL = Health-related quality of life

CRQ = Chronic Respiratory Questionnaire

HADS = Hospital Anxiety Depression Score

FT = Feeling Thermometer

## Authors' contributions

MP drafted and revised the manuscript. All authors participated in development of research protocols and in the design of the study. MP, CZ and HS resolved statistical and methodological issues. All authors read and corrected draft versions of the manuscript and approved the final manuscript.

## Pre-publication history

The pre-publication history for this paper can be accessed here:


